# Transcranial direct current stimulation (tDCS) enhances internal source monitoring abilities in healthy participants

**DOI:** 10.1371/journal.pone.0257010

**Published:** 2021-09-16

**Authors:** Isabella Kusztrits, Lynn Marquardt, Kenneth Hugdahl, Marco Hirnstein

**Affiliations:** 1 Department of Biological and Medical Psychology, University of Bergen, Bergen, Norway; 2 NORMENT Norwegian Centre for Mental Health Disorders Research, University of Bergen and Haukeland University Hospital, Bergen, Norway; 3 Department of Radiology, Haukeland University Hospital, Bergen, Norway; 4 Division of Psychiatry, Haukeland University Hospital, Bergen, Norway; University of Minho, PORTUGAL

## Abstract

Source monitoring refers to the ability to identify the origin of a memory, for example, whether you remember saying something or thinking about it, and confusions of these sources have been associated with the experience of auditory verbal hallucinations (AVHs). Both AVHs and source confusions are reported to originate from dysfunctional brain activations in the prefrontal cortex (PFC) and the superior temporal gyrus (STG); specifically, it is assumed that a *hypo*active PFC and a *hyper*active STG gives rise to AVHs and source confusions. We set out to test this assumption by trying to mimic this *hyper*temporal*/hypo*frontal model in healthy individuals with transcranial direct current stimulation (tDCS): the inhibitory cathode was placed over the left PFC and the excitatory anode over the left dorsolateral STG. Participants completed a reality monitoring task (distinguishing between external and internal memory sources) and an internal source monitoring task (distinguishing between two or more internal memory sources) in two separate experiments (offline vs. online tDCS). In the offline experiment (n = 34), both source monitoring tasks were completed after tDCS stimulation, and in the online experiment (n = 27) source monitoring tasks were completed while simultaneously being stimulated with tDCS. We found that internal source monitoring abilities were significantly enhanced during active online tDCS, while reality monitoring abilities were unaffected by stimulation in both experiments. We speculate, based on combining the present findings with previous studies, that there might be different brain areas involved in reality and internal source monitoring. While internal source monitoring seems to involve speech production areas, specifically Broca’s area, as suggested in the present study, reality monitoring seems to rely more on the STG and DLPFC, as shown in other studies of the field.

## Introduction

Source monitoring, which is the ability to make attributions about the origin of a memory, is important for remembering past experiences and is crucial for everyday situations, for example recalling who asked you to pick them up at the airport [1,2]. Depending on the source of information, Johnson, Hashtroudi (1) proposed three categories: external source monitoring (i.e., distinguishing between two or more external sources), internal source monitoring (i.e., distinguishing between two or more internal sources), and reality monitoring (i.e., distinguishing between external and internal sources) [1,3,4].

Impairment of source monitoring has been associated with patients with schizophrenia and especially with auditory verbal hallucinations (AVHs) [5], but not for hallucination-prone individuals [6]. Patients show a tendency to attribute inner speech processes to external sources (i.e., a deficit in reality monitoring) [7], and a tendency towards classifying imagined thoughts as verbalized thoughts (i.e., a deficit in internal source monitoring) [8]. Both processes originate from vivid mental imagery [9] that is more likely to become misattributed as coming from the outside [1].

Especially the left superior temporal gyrus (STG) has been shown to be involved in both vivid imagery and during AVHs [10,11]. Sugimori, Mitchell (12) found that this region was involved when participants misattributed verbal information as coming from external sources, even though it was internally generated. This activity in the STG was correlated with the tendency to experience AVHs [12]. In healthy people, auditory cortical regions, including the STG, showed increased activity when they were listening to speech derived from external sources, while inner speech did not elicit increased activity in these regions [13,14]. On the other hand, source confusions were associated with reduced activation in the prefrontal cortex (PFC) both in healthy people and in patients with schizophrenia [14].

Mondino, Haesebaert [15] reported a reduction in source monitoring errors in patients with schizophrenia after applying transcranial direct current stimulation (tDCS). Patients received tDCS with the cathode placed over the left STG and the anode over the left dorsolateral prefrontal cortex (DLPFC). The rationale behind this treatment is that AVH—and thus also source monitoring errors—arise from *hyper*active speech perception areas in the temporo-parietal region and *hypo*active prefrontal areas involved in cognitive control [16–18]. The cathode and anode are then meant to inhibit the *hyper*active speech perception and boost the *hypo*active cognitive control region [18], respectively. The study by Mondino, Haesebaert (15) thus further implicates an involvement of the STG and DLPFC in source monitoring.

Both regions have also been studied in healthy populations using tDCS. Mondino, Poulet [19] examined the left STG and DLPFC separately, by placing a larger reference electrode on the right occipital cortex which would lead to a more focal stimulation from the smaller electrode [20]. The authors found that anodal tDCS over the left temporo-parietal junction (TPJ; which is part of the STG) resulted in a higher likelihood to misattribute internally generated speech to externally perceived speech (i.e., reality monitoring). There was no tendency to misattribute self-generated thoughts to self-generated speech (i.e., internal source monitoring). These findings suggest a key role of the left TPJ in reality monitoring, but it does not seem to be involved in internal source monitoring. Cathodal tDCS over the left PFC did not lead to any modulation of source monitoring abilities, neither internal source monitoring nor reality monitoring [19]. However, the *hyper*temporal/*hypo*frontal model that was used in the treatment study by Mondino, Haesebaert (15) was not tested directly, as both regions were stimulated separately.

Another study examined the involvement of the right anterior medial PFC with tDCS [21]. Participants completed a reality monitoring task three times: real tDCS with the cathode placed over the right medial PFC and the anode over the left STG; sham tDCS with the same montage; and real tDCS with the cathode placed over the right medial PFC and the anode over the left visual area (as a control site). In the first of two separate experiments, participants received tDCS during the encoding stage, when they were asked to remember words. In the second experiment they received tDCS during the testing stage, when they were asked to indicate whether they had heard or imagined a word. Neither experiment found an effect of tDCS as compared to sham on reality monitoring. While some authors suggest that cathodal tDCS rarely induces inhibitory effects in cognitive tasks [22,23], the different findings of Mondino, Poulet (19) and Moseley, Mitrenga (21) might be due to differences in current strength, electrode montage (i.e., on the same hemisphere vs. on contralateral hemispheres), or timing (i.e., whether tDCS was given during encoding or testing stage).

Taken together, studies with healthy individuals and with patients who experience AVHs suggest that source monitoring processes require the involvement of the PFC and STG, which are also relevant in the occurrence of AVHs. But while there are efforts to reduce AVH [24] and at the same time strengthen source monitoring abilities with tDCS [15], little is known about the concrete brain mechanisms that underly the specific electrode montage used in these treatment studies. Moreover, neuroimaging studies in patients with schizophrenia and AVHs focus on source monitoring in general, with no explicit distinction between reality monitoring and internal source monitoring [4], and tDCS studies find inconsistent results between these two types [19,21]. Therefore, the aim of our study was to test the *hyper*temporal/*hypo*frontal model directly, by placing the anode over the left STG and the cathode over the left DLPFC in healthy individuals—to mimic the activity pattern that is assumed to underly AVHs and source monitoring deficits in schizophrenia patients. We carried out two experiments: an *offline experiment*, where source monitoring was carried out after stimulation with tDCS; and an *online experiment*, where source monitoring was carried out simultaneously with tDCS. The offline experiment was mostly exploratory in nature and motivated by findings that active stimulation modulates brain activity differently on a neural [22] and behavioural level [23], when stimulating offline or online. Even though the after-effects of active tDCS can last for over an hour, the simultaneous stimulation of brain regions that are involved in a specific task induces neuronal changes that affect the processing of information related to this task [24]. Therefore, we expected stronger behavioural effects of active tDCS on both internal source monitoring and reality monitoring abilities in the online experiment compared to the offline experiment.

## Methods and materials

### Participants

In total, we included 61 participants in two separate experiments (offline, n = 34; online, n = 27), with a mean age of 25.13 years (SD = 4.53). Participants in the offline experiment were part of a larger study with the aim to examine the underlying neuronal effects of tDCS on dorsolateral prefrontal and temporo-parietal areas during dichotic listening described elsewhere [25]. We excluded four participants from the original dataset, due to missing source monitoring data. We used flyers and word-of-mouth at the Haukeland University Hospital, Bergen, Norway, to recruit participants for both experiments. Participants in the online experiment were recruited separately for the purpose of this study.

In both experiments, exclusion criteria were past/present neurological or psychological disorders, head trauma, metallic implants, epilepsy in first degree relatives, pregnancy, claustrophobia, acute consumption of drugs or alcohol at time of testing, and severe skin diseases in the area of the electrode placement. In addition, all participants were screened for hearing impairment. Participant characteristics can be found in Table 1.

**Table 1:** Participant characteristics in both experiments.

	*Offline experiment* (*n* = 34)	*Online experiment* (*n* = 27)		
	M [26]	M [26]	*U*	*p*
**Age**	26.47 (4.76)	23.44 (3.65)	252.5	.002
**Education in years**	15.9 (1.99)	14.78 (2.54)	345.5	.097
**Days between tDCS sessions**	8.21 (3.29)	7.07 (1.64)	395.5	.334
	Absolute numbers (%)	Absolute numbers (%)	χ^2^_(1)_	*p*
**Gender**:			.898	.343
**Female**	16 (47.1%)	16 (59.3%)		
**Male**	18 (52.9%)	11 (40.7%)		
**Handedness**:			.006	.937
**Left-handed**	4 (11.8%)	3 (11.1%)		
**Right-handed**	30 (88.2%)	24 (88.9%)		
**Daily nicotine users (snuff)**	7 (20.6%)	7 (25.9%)	.242	.622

Notes. U = Mann-Witney U-test, χ^2^ = Chi-Square test, p = p-value.

All participants gave written informed consent in accordance with the Declaration of Helsinki [27] and were reimbursed for their participation. The study was approved by the Regional Committee for Medical Research Ethics in Western Norway (REK Vest) # 2013/2342.

#### Questionnaires

We collected basic demographic information including years of education and nicotine use. Participants’ handedness was measured with the Edinburgh Handedness Inventory [28], which provides a laterality score on a continuum from -100 (exclusively left handed) to +100 (exclusively right handed). With a cut-off score of +/- 50, 88.2% and 88.9% were right-handed in the offline and online experiment, respectively. This ratio between right- and left-handers in both experiments is in line with literature that suggests that roughly 10% of a randomly drawn sample are left-handers [29]. No participant had a laterality score between -50 and +50. The proportion of left-handers with typical left-hemispheric language specialization is roughly 70% as compared to 95% in right-handers [30]. Thus, while left-handers show a more variable lateralisation [31], the absolute number of atypically lateralized left-handers (~ 3%) is similar to the number of atypically lateralized right-handers (~ 4.5%) in a random sample with 90% right- and 10% left-handers [30]. Therefore, we decided to include both right- and left-handers. Common adverse side effects (e.g., headache, nausea) were measured with the tDCS Adverse Effects Questionnaire [32] after both sham and active tDCS sessions.

#### Source monitoring task

Internal source monitoring and reality monitoring was assessed using two Norwegian source memory tasks based on Keefe, Arnold [33] as well as Brunelin, Combris (5). Words presented in both tasks were contemporary Norwegian words consisting of one syllable and emotional neutral valence. More specifically, we first selected the most used one-syllable nouns in Norwegian, taken from the “NoWaC”, a large web-based corpus of Bokmål Norwegian [34]. Then, words were rated in an online survey by participants according to emotional valence, using the “self-assessment manikin scale” [35]. These resulted in 91 neutral words, which were randomly assigned to the different tasks. We excluded positive and negative words, as emotional stimuli seem to be processed differently in reality monitoring and internal source monitoring [36]. The words for the Hear-Imagine task were spoken by a native Norwegian female. For each task, we created two versions (Version A, and Version B), with a different set of 24 words to avoid learning effects between the first and the second tDCS session.

Each task was divided in a presentation phase and a test phase. In the presentation phase, 16 words were presented on a computer screen preceded by an instruction (both the instruction and the word were presented for 3 seconds). In the internal source monitoring task (Say-Imagine), participants were instructed to either “Say the following word aloud” (eight words) or “Imagine yourself saying the following word aloud” (eight words). In the reality monitoring task (Hear-Imagine), participants were instructed to “Imagine yourself hearing the following word” (eight words) or “Listen to the following word” (eight words). In the test phase, all 16 words were presented again in a randomised order, including eight distractor words that were not included in the presentation phase. In the test phase of the internal source monitoring task (Say-Imagine), participants were asked to indicate via button press for each word whether they had said the word, had imagined saying it, or whether it was a new word. In the reality monitoring task (Hear-Imagine), they indicated whether they had heard the word, imagined hearing the word, or whether it was a new word. Before each task started, there was a training phase with a maximum of four words. In total, both source monitoring tasks lasted approximately ten minutes.

Both tasks were administered separately. For example, when a participant would do the Hear-Imagine task first, they would start with the presentation phase, followed by the test phase of the Hear-Imagine task, and then the presentation and test phase of the Say-Imagine task. The type (Hear-Imagine/Say-Imagine) and version (A/B) of the source monitoring tasks were counterbalanced across participants and sessions in both experiments. In the offline experiment, 16 participants started with the Hear-Imagine task (9 with version A, 7 with version B), and 17 participants started with the Say-Imagine task (8 with version A, 8 with version B) in session 1. In the online experiment, 13 participants started with the Hear-Imagine task (7 with version A, 6 with version B), and 15 participants started with the Say-Imagine task (7 with version A, 8 with version B) in session 1.

Variables of interest were the number of misattributions of words that participants had imagined saying/hearing but indicated later that those words were actually said or heard (subsequently referred to as ‘externalization bias’; range: 0–8), the number of misattributions of words that participants had actually said/heard but indicated later they had imagined saying/hearing those words (subsequently referred to as ‘internalization bias’; range: 0–8), and the number of incorrectly identified ‘distractors’ (range: 0–8). In addition, we calculated old-new recognition accuracy, calculated by the number of correctly identified words minus the number of incorrectly identified words, as described in Garrison, Moseley (6) and Moseley, Mitrenga (21), which indicates the general recognition memory ability of participants.

#### tDCS

In both the offline and online experiment, participants were tested in two sessions, once with real stimulation and once with sham stimulation, in a double-blind design. The order of real/sham stimulation was counterbalanced. In the offline experiment, 18 participants received sham tDCS and 16 participants received real tDCS in session 1. In the online experiment, 14 participants received sham tDCS and 13 participants received real tDCS in session 1. A reporting checklist with an overview of the study’s design, following the recommendations by Buch, Santarnecchi [37] can be found in the supplementary materials.

**Fig 1:**
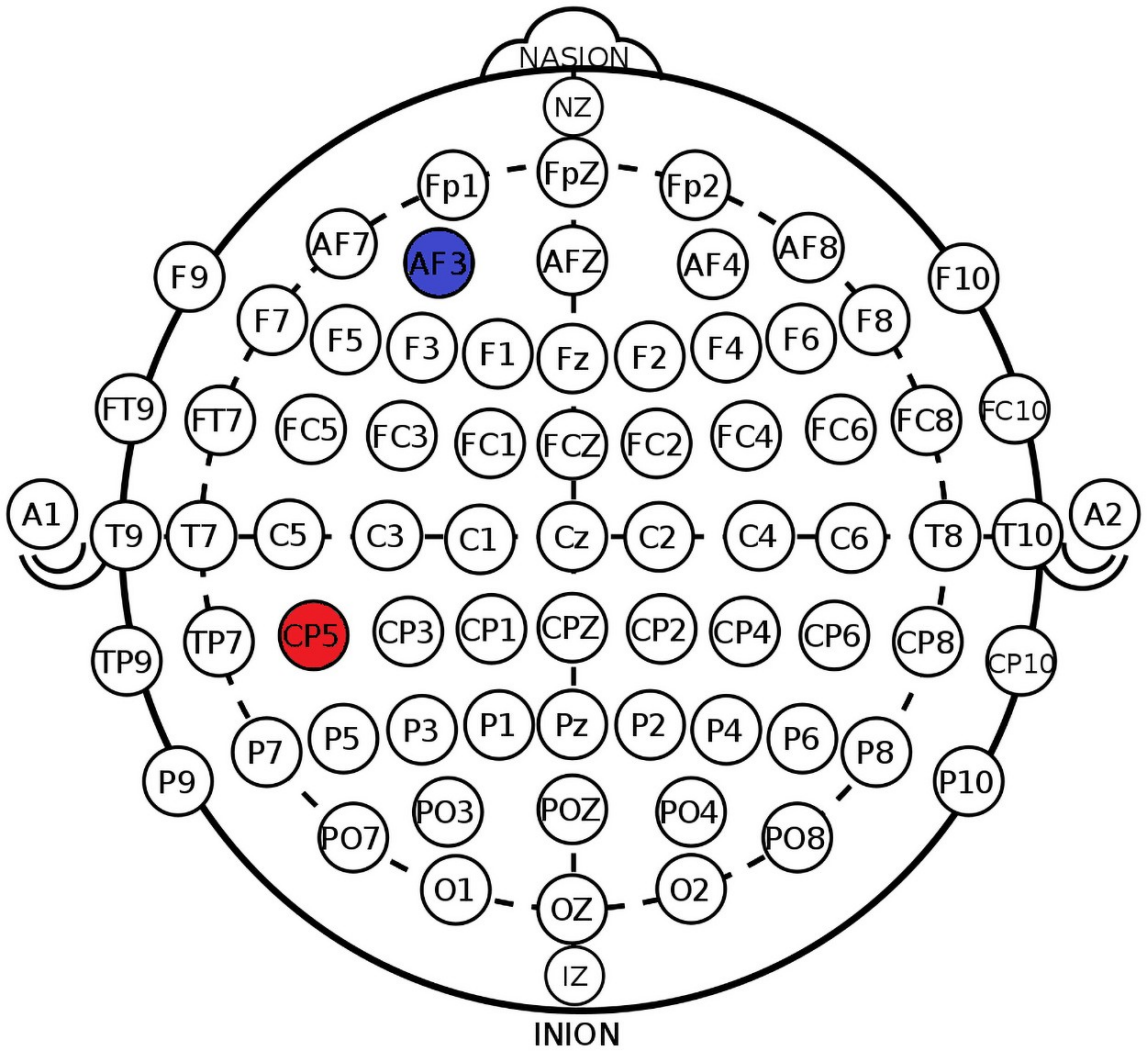
tDCS electrode placement with the cathode over AF3, the anode over CP5.

tDCS was delivered with a neuroConn DC-stimulator Plus (neuroConn GmbH, Ilmenau, Germany) and electrode positions were located with EEG caps (EASYCAP GmbH, 82211 Herrsching, Germany), based on the 10/20 system. The cathode and anode were placed over AF3 (left DLPFC) and CP5 (left STG), respectively (see Fig 1), and attached to the scalp via a rubber band. Real stimulation lasted 20 minutes (+30s ramp up and 30s ramp down) at 2mA (current density = 0.057mA/cm), and sham tDCS was delivered for 40s, followed by very weak pulses of 110μA lasting 15ms, provided every 550ms as an impedance check. Double-blinding was ensured by codes that determine whether a participant was stimulated with real or sham tDCS. These codes were assigned by a person who was not involved in the study. In the *offline experiment*, rectangular, MR compatible tDCS electrodes made of rubber (5cm x 7cm) were used. Electrodes were coated with conductive paste Ten20 (Weaver and Company, Aurora, United States of America) and a 9mg/ml NaCl solution to decrease impedance. Impedance was kept below 14.2kΩ, which was tested outside the MR scanner. For more details see also Marquardt, Kusztrits (25). In the *online experiment*, we used non-MR rubber electrodes (also 5cm x 7cm), that were placed into saline-soaked sponges. Electrode gel was used to decrease impedance between sponges and the skin.

#### Procedure—Offline experiment

In both sessions, participants completed a questionnaire regarding tDCS and functional magnetic resonance imaging (fMRI) safety before the electrodes were mounted and participants entered the scanner. They underwent structural MR, MR spectroscopy, and one fMRI sequence. During fMRI, they received tDCS while simultaneously performing a dichotic listening task. In the beginning of session 1, demographic information was collected, and a hearing test was performed. The whole MR session took approximately 40 min (for more details on the MR experiment see (Marquardt, 2020 #1172@@author-year)). Immediately after leaving the MR scanner and removing the tDCS electrodes, participants completed the tDCS adverse effects questionnaire and both source monitoring tasks separately. All participants finished both source monitoring tasks in a time window of 20 minutes after tDCS terminated. After the second source monitoring task in session 2, participants were asked to guess when they received real tDCS, to control for blinding.

#### Procedure—Online experiment

In Session 1, participants completed questionnaires about demographic information and did a hearing test after giving written consent. In both sessions, participants completed a questionnaire regarding tDCS safety before the electrodes were mounted. tDCS was applied during the whole task. Participants began the first source monitoring task five minutes after stimulation had started. There was no break between presentation phase and test phase. All participants finished the tasks before tDCS terminated and just waited quietly. Afterwards, the tDCS adverse effects questionnaire was completed. At the end of session 2, participants guessed in which session they received active tDCS.

#### Statistics

All analyses were conducted using IBM SPSS v.25 for Windows (IBM Corporation, Armonk, NY, USA).

We compared participants in both experiments (offline vs. online) regarding different characteristics. Gender and handedness were compared, using Chi^2^ tests. Age, years of education, and the time difference between session 1 and session 2 was compared, using Mann-Whitney U tests.

We performed in total six 2 x 2 repeated-measures analyses of variance (ANOVAs) with Stimulation (real vs. sham) as within- and Experiment (online vs. offline) as between-participants factors; one ANOVA for each source monitoring task (Say-Imagine, Hear-Imagine) and each dependent variable (internalization bias, externalization bias, incorrect distractors, old-new recognition accuracy) separately. The effect-size partial eta-squared (*η*_*p*_^*2*^) is provided, with *η*_*p*_^*2*^ = 0.01 representing a small effect, *η*_*p*_^*2*^ = 0.06 a medium effect, and *η*_*p*_^*2*^ = 0.14 a large effect [38]. Post-hoc paired samples t-tests were performed with Bonferroni-adjustment to correct for multiple testing [38] and descriptive means (instead of estimated marginal means) are given. To determine if baseline performances were comparable across the two experiments, we calculated paired samples t-tests between the sham conditions in the offline and online experiment.

A G*Power [39,40] analysis suggested that to obtain a significant Stimulation*Experiment interaction with n = 61, a minimum effect size of *η*_*p*_^*2*^ = 0.18 was required (with the settings: power = 0.8, α = 0.05, number of groups = 2, number of measurements = 2, correction among repeated measures = 0.5, non-sphericity correction = 1).

We calculated binomial tests with the test proportion 0.5, separately for each experiment, to examine whether the blinding worked. That is, if participants’ guesses when they received real stimulation was significantly different from 50% chance level. Adverse effects between real and sham tDCS were compared using Wilcoxon signed rank tests.

## Results

### Participant characteristics

Chi^2^ tests and Mann-Whitney-U tests showed no significant differences regarding gender, handedness, nicotine usage, years of education, and days between session 1 and session 2. Participants in the offline experiment were significantly older than in the online experiment (Table 1).

### Say-Imagine

#### Externalization bias

We found significant main effects for *stimulation* (*F*_*(1*,*59)*_ = 33.33, *p* < .01, *η*_*p*_^*2*^ = .36) and *experiment* (*F*_*(1*,*59)*_ = 7.85, *p* = .01, *η*_*p*_^*2*^ = .12). Also, the interaction effect stimulation*experiment was significant (*F*_*(1*,*59)*_ = 12.71, *p* = .01, *η*_*p*_^*2*^ = .18). As can be seen in Table 2, none of the participants in the *online experiment* that received real stimulation made a single mistake (both mean and standard deviation is zero). Post-hoc t-tests showed significant results between real vs. sham tDCS in the *online experiment* (*t* = -7.19, *p* < .01), but not in the *offline experiment* (*t* = -1.52, *p* = .14). Sham tDCS did not differ between the *offline* and *online experiment* (*t* = 0.12, *p* = .91).

**Table 2:** Means and standard deviations for total number of errors as well as means of error rates in percent in the Say-Imagine task.

	externalization bias		internalization bias		incorrect distractors		old-new recognition accuracy	stimulation condition
	M [26]	%	M [26]	%	M [26]	%	M [26]	
**offline**	1.27 (1.29)	15.88	1.09 (1.33)	13.63	1.06 (0.98)	13.25	13.29 (4.43)	real
	1.68 (1.20)	21.00	1.06 (0.85)	13.25	1.06 (1.10)	13.25	13.29 (5.37)	sham
**online**	0.00 (0.00)	0	0.00 (0.00)	0	1.15 (1.13)	14.38	15.52 (4.66)	real
	1.74 (1.26)	21.75	1.48 (1.05)	18.50	1.56 (1.37)	19.50	8.96 (7.00)	sham

Notes. Maximum possible number of absolute errors was 8.

#### Internalization bias

We found significant main effects for *stimulation* (*F*_*(1*,*59)*_ = 7.42, *p* = .01, *η*_*p*_^*2*^ = .11) and *experiment* (*F*_*(1*,*59)*_ = 9.65, *p* = .01, *η*_*p*_^*2*^ = .14). Moreover, the interaction effect stimulation*experiment was significant (*F*_*(1*,*59)*_ = 6.68, *p* = .01, *η*_*p*_^*2*^ = .10). Again, the interaction arose because participants in the *online experiment* did not commit a single mistake when receiving real tDCS (see Table 2). Post-hoc t-tests showed significant results between real vs. sham tDCS in the *online experiment* (*t* = -7.32, *p* < .01), but not in the *offline experiment* (*t* = 0.13, *p* = .90). Sham tDCS did not differ between the *offline* and *online experiment* (*t* = -2.01, *p* = .06).

#### Incorrect distractors

Neither the main effects *stimulation* (*F*_*(1*,*59)*_ = 0.98, *p* = .33, *η*_*p*_^*2*^ = .02) and *experiment* (*F*_*(1*,*59)*_ = 1.94, *p* = .17, *η*_*p*_^*2*^ = .03), nor the interaction stimulation*experiment became significant (*F*_*(1*,*59)*_ = .98, *p* = .33, *η*_*p*_^*2*^ = .02).

#### Old-new recognition accuracy

We found a significant main effect for *stimulation* (*F*_*(1*,*59)*_ = 16.04, *p <* .01, *η*_*p*_^*2*^ = .21), but not for *experiment* (*F*_*(1*,*59)*_ = 0.88, *p* = .35, *η*_*p*_^*2*^ < .02). Also, the interaction effect stimulation*experiment was significant (*F*_*(1*,*59)*_ = 16.04, *p <* .01, *η*_*p*_^*2*^ = .21). Post-hoc tests showed a significant result between real vs. sham tDCS in the *online experiment* (*t* = 5.04, *p* < .01), but not in the *offline experiment* (*t* = 0.00, *p* = 1.00). Sham tDCS did differ significantly between the *offline* and *online experiment* (*t* = 2.58, *p* = .02).

### Hear-Imagine

#### Externalization bias

There was neither a significant main effect for stimulation (F(1,59) = 1.05, p = .31, ηp2 = .02) nor for experiment (F(1,59) = .28, p = .60, ηp2 = .01). Also, the interaction effect stimulation*experiment was not significant (F(1,59) = 1.32, p = .26, ηp2 = .02). Means and standard deviations can be found in Table 3.

**Table 3:** Means and standard deviations of absolute errors as well as means of the error rates in percent in the Hear-Imagine task.

	externalization bias		internalization bias		incorrect distractors		old-new recognition accuracy	stimulation condition
	M [26]	%	M [26]	%	M [26]	%	M[26]	
**offline**	1.65 (1.41)	20.63	1.09 (0.97)	16.63	0.68 (1.04)	8.50	13.29 (5.19)	real
	1.68 (1.53)	21.00	1.12 (1.20)	14.00	0.82 (0.76)	10.25	13.59 (4.51)	sham
**online**	2.07 (1.66)	25.88	1.30 (1.24)	16.25	0.93 (1.11)	11.63	9.56 (6.64)	real
	1.56 (1.16)	19.50	1.11 (1.01)	13.88	0.74 (1.06)	9.25	12.44 (5.47)	sham

Notes. Maximum possible number of absolute errors was 8.

#### Internalization bias

There was neither a significant main effect for *stimulation (F*_*(1*,*59)*_ = .16, *p* = .69, *η*_*p*_^*2*^ = .01) nor for *experiment* (*F*_*(1*,*59)*_ = .23, *p* = .64, *η*_*p*_^*2*^ = .01). Also, the interaction effect stimulation*experiment was not significant (*F*_*(1*,*59)*_ = 0.31, *p* = .58, *η*_*p*_^*2*^ = .01).

#### Incorrect distractors

There was neither a significant main effect for *stimulation* (*F*_*(1*,*59)*_ = .02, *p* = .89, *η*_*p*_^*2*^ < .01) nor for *experiment* (*F*_*(1*,*59)*_ = .15, *p* = .70, *η*_*p*_^*2*^ = .01). Also, the interaction effect stimulation*experiment was not significant (*F*_*(1*,*59)*_ = 1.48, *p* = .23, *η*_*p*_^*2*^ = .02).

#### Old-new recognition accuracy

We found no significant main effect for *stimulation (F*_*(1*,*59)*_ = *3*.*48*, *p* = .07, *η*_*p*_^*2*^ = .06), but for *experiment* (*F*_*(1*,*59)*_ = *4*.*84*, *p* = .03, *η*_*p*_^*2*^ = .08). Also, the interaction effect stimulation*experiment was not significant (*F*_*(1*,*59)*_ = *2*.31, *p* = .13, *η*_*p*_^*2*^ = .04).

### Covariates

Because nicotine has been reported to affect tDCS outcome [41], and the participants in the offline experiment were significantly older than in the online experiment, we re-ran the ANOVAs with nicotine consumption and age as additional covariates. However, including the covariates did not change the pattern of significant and non-significant effects reported above.

### Blinding and adverse effects

In the *offline* and *online experiment*, 47% and 56% of participants guessed correctly when they received real stimulation, respectively. A binomial test found no statistical difference from 50% chance level in both experiments (*offline*: *p* = .86; *online*: *p* = .70).

Wilcoxon signed-rank tests showed no significant difference in the frequency of common adverse side effects between real and sham tDCS in either experiment. Z-scores and p-values can be found in the supplementary materials.

## Discussion

We examined internal source monitoring and reality monitoring abilities in healthy participants with the anode and cathode placed over the left STG and DLPFC, respectively. While tDCS had no effect on reality monitoring, neither in the offline nor in the online experiment, internal source monitoring was significantly improved in all measures with real tDCS as compared to sham in the online experiment. Participants showed higher general recognition ability, and made both fewer externalization and internalization errors, while the number of incorrectly identified distractors was similar.

### Internal source monitoring

So far, the underlying neuronal mechanisms for *internal* source monitoring were unclear. While inhibiting activity in the temporo-parietal cortex with repetitive transcranial magnetic stimulation led to both reduced frequency of AVHs and increased internal source monitoring abilities in patients with schizophrenia [42], internal source monitoring was unaffected in healthy individuals when being stimulated with anodal tDCS in the STG region [19]. In contrast, our study showed a clear modulation of internal source monitoring effects with the anodal STG and cathodal DLPFC montage.

A possible explanation for these inconsistent results may be provided by another study from our group [25], published after we had completed data collection for the present study, which found (a) no excitatory/inhibitory effect underneath the electrodes in the STG/DLPFC region with the ipsilateral frontotemporal montage and (b) that the electrical field was strongest in the left central sulcus region/Broca’s area—between the two electrodes. Thus, the modulation of internal source monitoring effects observed in the present study might result from an indirect stimulation of Broca’s area. This may not be surprising given that Broca’s area is also involved in lexical, grammatical, and phonological processing [43], and verbal working memory [44]. In addition, Flinker, Korzeniewska [45] reported in their study that Broca’s area specifically has a mediating role in speech production, by sending articulatory codes that originate from temporal areas (where neural representations of words are created) to the motor cortex (where these articulatory codes are turned into gestures). The excitation of neurons specialized for speech production in our study might have led to an enhanced ability to differentiate between imagined and spoken words.

In addition, Chen, Mathalon [46] suggested that Broca’s area is also involved in a process called auditory corollary discharge. This mechanism is hypothesized to facilitate the distinction of one’s own speech from externally generated sounds. A copy of motor commands, transferred from speech production regions in the frontal lobes to the auditory cortex, produces an expectancy of consequences in the auditory cortex to prepare it for an imminent self-generated speech sound. This provides a mechanism for recognizing these sounds as their own by minimizing the auditory cortical response to these self-generated sounds [47]. When we assume that Broca’s area was most affected by the stimulation protocol of the present study, it seems plausible that tDCS could have an effect on corollary discharge and reduce source misattributions. An involvement of Broca’s area in internal source monitoring would also explain why both Mondino, Poulet (19) and Moseley, Mitrenga (21) did not report any changes in internal source monitoring after tDCS in healthy individuals. Mondino, Poulet (19) targeted specifically the PFC and STG using large reference electrodes over the occipital lobe. Similarly, Moseley, Mitrenga (21) specifically targeted the PFC and STG with an interhemispheric electrode montage, thus placing the electrodes further away than with the current fronto-temporal montage. Interestingly, Mondino, Haesebaert (15) used a similar frontotemporal electrode montage as in the present study, but with a reversed cathode-anode placement, and found a reduction in source monitoring errors as well.

It is important to note that the old-new recognition accuracy was modulated, as well as both an externalizing and an internalizing bias was induced by tDCS in the present study—but no modulation of the distractor words. If the number of distractor words had also been affected, this would have been indicative of a more general effect of tDCS on memory processes. The fact that distractor words were not affected further supports the notion that Broca’s area might be involved in internal source monitoring processes.

### Reality monitoring

Mondino, Poulet (19) found a modulatory effect of anodal tDCS in the left STG (with a reference electrode above the occipital cortex), where stimulation increased the number of errors (i.e., imagined words were incorrectly recognized as heard) in *reality monitoring* abilities. Provided that we mainly stimulated Broca’s area with our ipsilateral, frontotemporal montage in the present study, our findings on reality monitoring would thus be in line with Mondino, Poulet (19). Moseley, Mitrenga (21), on the other hand, did not find any effects of real tDCS on reality monitoring, also not on old-new recognition accuracy, but they used a contralateral frontotemporal electrode setup and a lower electrical current than in the present study and the study by Mondino, Poulet (19) (i.e., 1 mA vs. 2 mA). Moseley, Mitrenga (21) suggest that the PFC plays a less important role in reality monitoring than previously assumed, as this region shows increased activation during retrieval of source information, with a focus on monitoring self-generated information [4] and acting as a gateway between task relevant and task independent thought [48]. Moseley, Mitrenga (21) argued that this activation is valid for related processes used during reality monitoring, but it is not causally necessary for encoding or source judgements. Taken together, the results from tDCS studies in healthy participants point to the involvement of at least the left STG in reality monitoring.

### Offline vs. online tDCS

As adverse effects did not differ between the offline and online experiment, and the guessing of whether they received real or sham tDCS was not above chance level, we can assume that the blinding of real and sham stimulation conditions worked, and that participants’ expectations regarding real/sham stimulation did not affect the results. Similar results regarding blinding were reported by other authors who specifically investigated the differences between real and sham stimulation in tDCS [49,50].

We also compared the sham conditions between the offline and online experiment regarding internal source monitoring to determine if there was a difference in the baseline performance between the two experiments. The results were ambivalent: There were no differences in sham conditions for the internalization and externalization bias. However, the old-new recognition accuracy of internal source monitoring abilities was significantly higher during sham in the offline as compared to during sham in the online experiment. This could hint that the offline design made old/new recognition a bit easier for participants or that the offline sample in general performed a bit better in terms of old/new recognition. However, it does not change the finding that internal source monitoring was affected by real versus sham tDCS in the online experiment.

Another result was that we only found a modulatory effect of source monitoring in the online, but not the offline experiment. This has previously been demonstrated by other authors [47,51], who reported that timing tDCS with motor learning and cognitive training tasks simultaneously (i.e., online tDCS) yielded better performance outcomes than offline tDCS.

### Limitations

The results in the offline experiment could have been confounded by the participants being tired after the scanning protocol or potentially less motivated to perform the source monitoring task. Secondly, participants in the offline experiment were performing an acoustic language task [i.e., dichotic listening, [16] in the MR scanner while receiving tDCS [25], which might have led to a different outcome as compared to the online experiment. Thirdly, there was a break of roughly 20 minutes between the termination of tDCS and the end of the source monitoring task, in which two more MR-sequences were applied and electrodes had to be removed from the scalp. This timespan might have reduced the impact of tDCS on source monitoring tasks. Finally, participants in the offline experiment were significantly older than in the online experiment. However, given the means were 26 and 23 years, respectively, we find it hard to believe that this could account for the different outcomes of the experiments.

Another potentially limiting factor in general, is the relatively few discrete responses (16 per source monitoring task, eight per dependent variable) and the relatively few mistakes (range: 0–25%). Thus, our findings rest on relatively few events, which could lead to ceiling effects. As pointed out by Moseley, Mitrenga (21) the difficulty could be increased by introducing a break between the encoding and testing phase. However, that would make it difficult to include both phases in a single tDCS period. For the same reason, increasing the number of trials would make it difficult to complete both tasks in the short stimulation window. As one of our goals was to corroborate the findings of Mondino, Poulet (20) we opted for similar tasks and experimental designs. We tried to compensate for the few trials by testing a reasonable number of participants, given that this was a tDCS-study with a repeated design.

Finally, although with n = 61 the sample size is relatively large compared to other studies in the field, we only had sufficient power (.80) to detect large effects. It is thus possible that we may have missed smaller effects.

## Conclusion

In summary, the current study is in line with previous findings that tDCS over prefrontal and temporo-parietal areas affects source monitoring in healthy participants, in general. Beyond the existing literature, however, we tentatively interpret the findings such that reality monitoring and internal source monitoring involve different brain structures: While reality monitoring may be more specifically linked to (left) prefrontal and temporo-parietal areas, internal source monitoring rather seems to involve (left) Broca’s area. However, this needs to be confirmed by future studies.

## Supporting information

S1 Table Reporting checklist for tDCS studies. Variant appropriate to our study marked with “X”.
(DOCX)

S2 Table Frequency of adverse events during sham and real tDCS session in the offline experiment. (DOCX)

S3 Table Frequency of adverse events during sham and real tDCS session in the online experiment. (DOCX)
