# Supplementary material for: Transcranial direct current stimulation (tDCS) enhances internal source monitoring abilities in healthy participants
Source: PLoS One. 2021 Sep 16;16(9):e0257010. doi: 10.1371/journal.pone.0257010 (PMC8445448; doi:10.1371/journal.pone.0257010)
Supplement: S1 Table — Variant appropriate to our study marked with “X”. (DOCX) [file pone.0257010.s001.docx]

S1 Table

Reporting checklist for tDCS studies. Variant appropriate to our study marked with “X”.

| Experimental Design Factors: | | | |
| --- | --- | --- | --- |
| Controls used | □ None | X Sham | □ Active |
| Blinding used | □ None | □ Single | X Double |
| Hypothesis statement | X Yes | □ No |  |
| If Hypothesis-based: |  |  |  |
| Power-analysis statement | X Yes | □ No |  |
| Pre-registration | □ Yes | X No |  |
| Exploratory-based | □ Yes | X No |  |
| Sample-size estimation (i.e. – power analysis) | □ Yes | X No |  |
|  |  |  |  |
| Participant Factors: | Reported? | Controlled? |  |
| Number of subjects | X | □ |  |
| Age of subjects | X | □ |  |
| Gender of subjects | X | □ |  |
| Handedness of subjects | X | □ |  |
| Subjects prescribed medication | □ | X |  |
| Use of CNS active drugs (e.g. anti-convulsants) | □ | X |  |
| Neuropsychological evaluation | □ | □ |  |
| Any medical conditions | □ | X |  |
| History of specific repetitive motor activity | □ | X |  |
| Years of Education completed | X | □ |  |

Continuation S1 Table 1

| Stimulation Factors: | Reported? | Controlled? |  |
| --- | --- | --- | --- |
| Scalp position of tDCS electrodes | X | □ |  |
| MRI-based localization of tDCS electrodes | □ | □ |  |
| Electrode type (size and geometry) | X | □ |  |
| Current density of applied stimulation | X | □ |  |
| Type of stimulator used (e.g. brand) | X | □ |  |
| Stimulation intensity | X | □ |  |
| Stimulation ramp time | X | □ |  |
| Stimulation duration | X | □ |  |
| Number of Sessions | X | □ |  |
| If Multiple Sessions: |  |  |  |
| Time interval between sessions | X | □ |  |
| Subject attention (level of arousal) during testing | □ | □ |  |
| Subject activities during stimulation | X | □ |  |
| tDCS-induced sensations (i.e. – itching, pain, heat, pinching, burning) | X | □ |  |
|  |  |  |  |
| Analysis & Statistics factors: | | | |
| Effect-size(s) reported | X Yes | □ No |  |
| Raw data uploaded to publicly accessible data repository | □ Yes | X No |  |
| Analyzed data uploaded to publicly accessible data repository | □ Yes | X No |  |
| Full analysis protocol including custom scripts uploaded to publicly accessible data repository | □ Yes | X No |  |
