# Supplementary material for: Transcranial direct current stimulation (tDCS) enhances internal source monitoring abilities in healthy participants
Source: PLoS One. 2021 Sep 16;16(9):e0257010. doi: 10.1371/journal.pone.0257010 (PMC8445448; doi:10.1371/journal.pone.0257010)
Supplement: S2 Table — (DOCX) [file pone.0257010.s002.docx]

S2 Table

Frequency of adverse events during sham and real tDCS session in the offline experiment.

|  | Real tDCS | | | | Sham tDCS | | | |  |  |
| --- | --- | --- | --- | --- | --- | --- | --- | --- | --- | --- |
| Adverse effects | Mild | Moderate | Severe | **∑** | Mild | Moderate | Severe | **∑** | Z | p-value |
| Headache | 7 | 1 | - | **8** | 8 | - | - | **8** | <.001 | 1.000 |
| Neck pain | 5 | 2 | - | **7** | 5 | 1 | - | **6** | -.378 | .705 |
| Scalp pain | 6 | 4 | - | **10** | 8 | 4 | - | **12** | -.707 | .480 |
| Tingling | 13 | 6 | - | **17** | 14 | 5 | - | **19** | <.001 | 1.000 |
| Itching | 6 | - | - | **6** | 7 | 2 | - | **9** | -.905 | .366 |
| Burning sensation | 9 | 4 | - | **13** | 6 | 4 | 1 | **11** | -1.134 | .257 |
| Skin redness | 11 | 1 | - | **12** | 9 | - | - | **9** | -1.134 | .257 |
| Sleepiness | 15 | 8 | 2 | **25** | 15 | 8 | 1 | **24** | -.378 | .705 |
| Trouble concentrating | 10 | 4 | - | **14** | 10 | 4 | 1 | **15** | -.447 | .655 |
| Acute mood change | 2 | - | - | **2** | 1 | - | - | **1** | -1.000 | .317 |
