# Supplementary material for: Transcranial direct current stimulation (tDCS) enhances internal source monitoring abilities in healthy participants
Source: PLoS One. 2021 Sep 16;16(9):e0257010. doi: 10.1371/journal.pone.0257010 (PMC8445448; doi:10.1371/journal.pone.0257010)
Supplement: S3 Table — (DOCX) [file pone.0257010.s003.docx]

S3 Table

Frequency of adverse events during sham and real tDCS session in the online experiment.

|  | Real tDCS | | | | Sham tDCS | | | |  |  |
| --- | --- | --- | --- | --- | --- | --- | --- | --- | --- | --- |
| Adverse effects | Mild | Moderate | Severe | **∑** | Mild | Moderate | Severe | **∑** | Z | p-value |
| Headache | 8 | 1 | - | **9** | 9 | - | - | **9** | <.001 | 1.000 |
| Neck pain | 4 | - | - | **4** | 1 | - | - | **1** | -1.342 | .180 |
| Scalp pain | 4 | 2 | - | **6** | 2 | 1 | 1 | **4** | -1.000 | .317 |
| Tingling | 8 | 9 | 1 | **18** | 11 | 6 | 3 | **20** | -.632 | .527 |
| Itching | 7 | 5 | 1 | **13** | 10 | 6 | 2 | **18** | -1.890 | .059 |
| Burning sensation | 8 | 3 | 1 | **12** | 13 | 4 | - | **17** | -1.508 | .132 |
| Skin redness | 6 | 1 | - | **7** | 6 | - | - | **6** | -.378 | .705 |
| Sleepiness | 4 | 3 | 1 | **8** | 6 | 2 | 1 | **9** | -.447 | .655 |
| Trouble concentrating | 5 | 3 | - | **8** | 7 | 1 | - | **8** | <.001 | 1.000 |
| Acute mood change | 1 | 1 | - | **2** | - | - | - | **-** | -1.414 | .157 |
